# The association between diet quality indices and oxidative stress biomarkers in male footballers and healthy active controls

**DOI:** 10.1186/s13104-024-06858-w

**Published:** 2024-07-15

**Authors:** Mahsa Zare, Zainab Shateri, Mahboobeh Shakeri, Mehran Nouri, Sahar Zare, Parvin Sarbakhsh, Mohammad Hassan Eftekhari, Bahram Pourghassem Gargari

**Affiliations:** 1 Student Research Committee, Faculty of Nutrition and Food Sciences, Tabriz University of Medical Sciences, Tabriz, Iran; 2 Department of Nutrition and Biochemistry, School of Medicine, Ilam University of Medical Sciences, Ilam, Iran; 3 Endoocrine and Metabolism Research Center, Isfahan University of Medical Sciences, Isfahan, Iran; 4 Health Research Institute, Babol University of Medical Sciences, Babol, Iran; 5 Health Policy Research Center, Institute of Health, Shiraz University of Medical Sciences, Shiraz, Iran; 6 Nursing Department, Eghlid Branch, Islamic Azad University, Eghlid, Iran; 7 Department of Statistics and Epidemiology, School of Public Health, Tabriz University of Medical Sciences, Tabriz, Iran; 8 Department of Clinical Nutrition, School of Nutrition and Food Sciences, Shiraz University of Medical Sciences, Shiraz, Iran; 9 Nutrition Research Center, Department of Biochemistry and Diet Therapy, Faculty of Nutrition and Food Sciences, Tabriz University of Medical Sciences, Tabriz, Iran

**Keywords:** Diet quality, Oxidative stress, Healthy eating index, Dietary Quality Index, F_2alpha_-isoprostane, 8-hydroxy-2'-deoxyguanosine, Football

## Abstract

**Objective:**

The aim of the present study was the association between the relationship between Dietary Quality Index-International (DQI-I) and Healthy Eating Index (HEI) and the urinary levels of F_2alpha_-isoprostane (F_2a_-IP) and 8-hydroxy-2’-deoxyguanosine (8-OHdG) was investigated as indicators of oxidative stress.

**Results:**

Based on HEI (low, moderate, and good), the diet quality of both groups was classified as moderate. In all participants, HEI (β=-0.29; *P =* 0.04) and DQI-I (β=-0.46; *P =* 0.005) were inversely associated with 8-OHdG. Furthermore, a negative correlation was found between HEI (mean β=-3.53; *P* = 0.04) and DQI-I (mean β=-5.53; *P* = 0.004) with F_2a_-IP. The quality of the footballers’ diet was higher than that of the control group. Following a high-quality diet, which is rich in antioxidants, is likely to effectively reduce oxidative stress.

## Introduction

Football is a team-based speed endurance sport with low-intensity movements and intense actions that include walking, jogging, standing, sprinting, turning, tackling, and jumping [1]. Muscle cells produce reactive oxygen species during exercise [2]. When exogenous and endogenous antioxidants cannot balance reactive oxygen species, the condition is called oxidative stress (OS) [3].

Exercise-induced OS can result in damage to deoxyribonucleic acid (DNA), proteins, and lipids, negatively affecting athletic performance and recovery [4]. Two reliable biomarkers of OS are 8-hydroxy-2’-deoxyguanosine (8-OHdG), a biomarker of DNA nucleobase modifications by hydroxyl radicals [5], and F_2alpha_-isoprostane (F_2a_-IP), a biomarker of peroxidation of arachidonic acid by free radicals [6]. Earlier studies have indicated that urinary excretions of F_2a_-IP and 8-OHdG are associated with factors such as gender, age, smoking, body mass index (BMI), exercise, and diet [7]. Increased concentrations of 8-OHdG have been implicated in several diseases including cardiovascular disease, diabetes, some cancers, etc. [5, 7, 8], and various methods are available for DNA damage screening [9, 10], and this biomarker is just one of them. Also, an increase in F_2a_-IP concentrations has been reported in diabetes, chronic kidney diseases, cardiovascular disease, some cancers, age-related diseases, etc. [7].

In sports, food is one of the main factors affecting health, performance, and recovery [11, 12]. Since food ingredients are consumed together in a usual diet, they may interact in inhibitory or synergistic ways. Therefore, the assessment of a whole diet appears more beneficial than the assessment of a single ingredient [13–15].

Diet quality is defined as an individual’s dietary adequacy and adherence to dietary guidelines [16, 17], which is also related to the risk of chronic diseases [18]. Diet quality provides a broader image of the whole diet (food and nutrient consumption). Therefore, measuring diet quality indices is useful for predicting the association between a whole diet and health consequences [13, 19]. It has been reported that oxidative balance is related to the quality of the diet [13, 20]. In this regard, to prevent the harmful effects of OS and subsequently improve the performance of footballers, they are encouraged to consume a high-quality diet with sufficient calories, macronutrients, and micronutrients [21, 22].

The Healthy Eating Index (HEI) and the Dietary Quality Index-International (DQI-I) are two common tools for assessing diet quality. The HEI-2015 (the latest version of the HEI) is based on the key recommendations of the 2015–2020 Dietary Guidelines for Americans [23, 24]. Additionally, the DQI-I was developed based on international dietary guidelines [25], and previous studies have demonstrated that the HEI and DQI-I can be used to evaluate the quality of the Iranian diet [26].

The importance of diet quality for football players has been indicated, as explained earlier [21, 22]. Additionally, to our knowledge, no research has been conducted on the relationship between diet quality and oxidative status among footballers. This makes it necessary to evaluate the diet quality of football players. Given the limited data, this study aimed to evaluate the HEI and DQI-I in male footballers and healthy active controls. Also, it sought to determine the relationships between the HEI and DQI-I with the concentrations of 8-OHdG and F_2a_-IP in the urine.

## Methods

### Study design, sample size, and population

The current research data were obtained from a research project, and some of the findings have been previously published [27–29]. The current descriptive-analytical study was conducted on 90 males in two groups (45 footballers and 45 active controls), and the groups were individually matched according to BMI and age. Sampling and data collection were carried out between April 2022 and July 2022.

G*Power software was used to estimate the sample size based on the mean difference in concentration of 8-OHdG in the urine between athlete and non-athlete groups, as provided by Rahimi et al.‘s research [30]. Due to the existing correlation between matched athlete and control groups, a paired-design method was performed considering 80% power, 95% confidence, and a 0.15 correlation between the concentration of 8-OHdG in athlete and control groups. The required sample size for each group (considering a 5% sample drop) was determined to be 45 people.

**Fig. 1:**
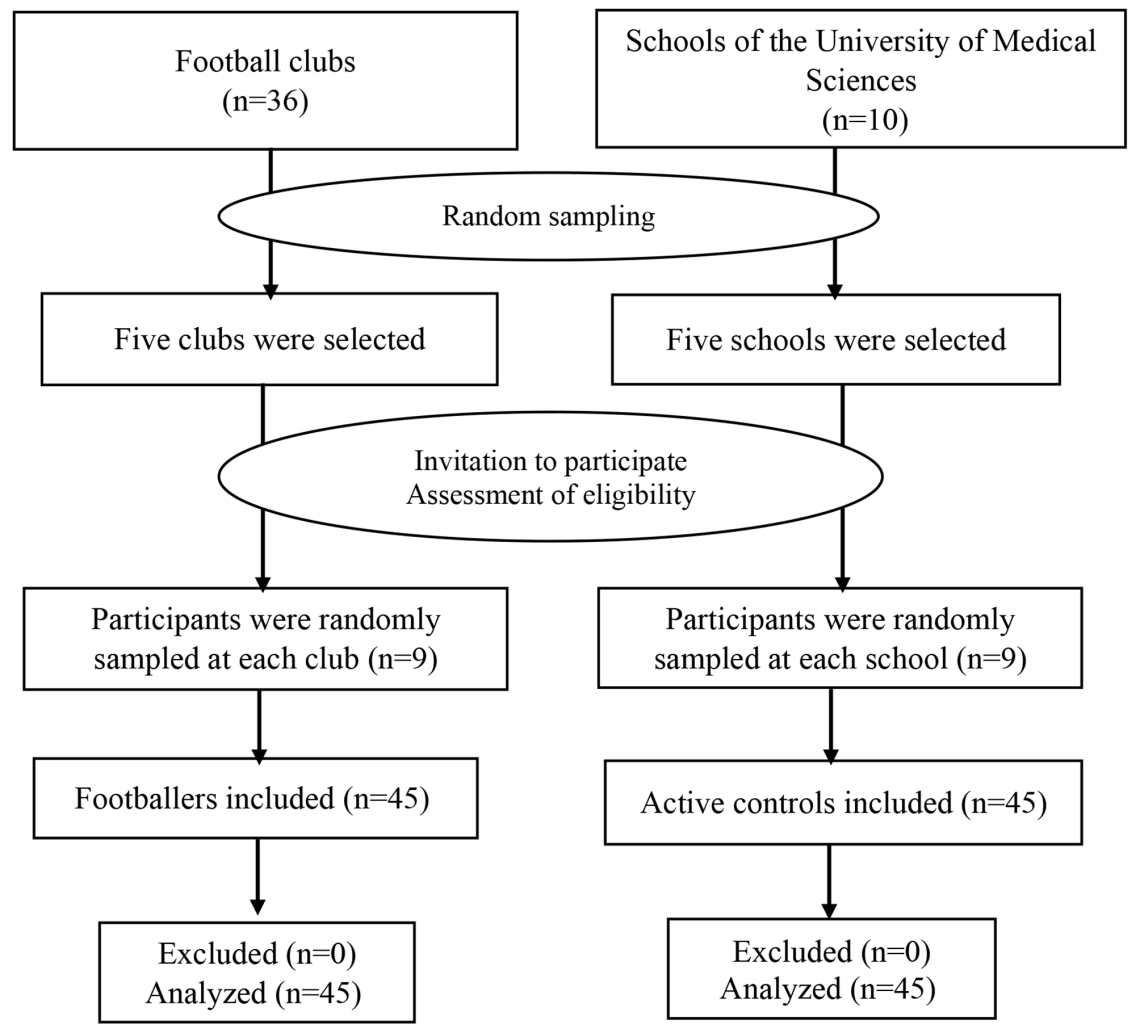
Flow chart of the study

Footballers were chosen using a cluster sampling procedure from 36 football clubs in Shiraz City, Iran. First, five clubs were randomly selected, and then nine eligible footballers from each selected club were invited. Similarly, a control group was recruited from ten schools affiliated with Shiraz University of Medical Sciences through a comparable procedure. Initially, five schools were randomly selected, and then, nine individuals were randomly included from each selected school. The flow chart of the participants’ recruitment is presented in Fig. 1.

### Inclusion and exclusion criteria

The inclusion criteria for footballers were as follows: (1) BMI of 20–25 kg/m2 and aged 20–30 years; (2) Football experience within the last 2–3 years with a training protocol of 3–4 days/week and 1.5–2 h/session; (3) Metabolic equivalent of task (MET) > 3000 (min/week); (4) No smoking or drinking alcohol; (5) Not taking drugs that alter the oxidants-antioxidants balance or antioxidant supplements in the past month; (6) Stable body weight and eating habits in the past two months. The eligibility criteria for the control group were as follows: individuals matched with footballers according to age and BMI, 600 < MET < 3000 (min/week), and meeting the criteria 4–6 mentioned above for the footballer group and items #4–6.

The exclusion criteria for all subjects included: (1) Subjects with cardiovascular, liver, respiratory, kidney, thyroid, and inflammatory diseases, stroke, malignancies, hyperlipidemia, hypertension, and diabetes; (2) Hormone therapy; (3) Consumption of nonsteroidal anti-inflammatory drugs (NSAIDs); (4) Those who completed less than 90% of the food frequency questionnaire (FFQ) items.

### Assessment of physical activity and anthropometric indices

All the variables were measured by a trained examiner to prevent any measurement errors. To assess physical activity (expressed as MET-hr/week), the International Physical Activity Questionnaire (IPAQ) was utilized [31]. A digital scale and a wall-mounted stadiometer (Seca, Hamburg, Germany) were used to measure weight and height with an accuracy of 0.1 kg and 0.1 cm, respectively. To estimate BMI (kg/m^2^), weight (kg) was divided by height (m^2^).

### Assessment of dietary intakes

A trained dietitian administered a valid semi-quantitative FFQ with 168 food items to evaluate participants’ dietary intake over the previous year [32]. Each participant reported his average intake; then, these reports were transformed into daily grams using household measures [33]. The content of nutrients and energy of each food item was calculated using Nutritionist IV software modified for Iranian foods (First Databank, San Bruno, CA, USA).

### Calculation of HEI

The HEI-2015 scores were calculated based on 13 components categorized into adequacy and moderation subgroups that were scored on a density basis out of 1000 calories (except for fatty acid, which is a ratio) with a maximum total score of 100 points. Nine adequacy components, including whole fruits, total fruit, whole grains, total vegetables, greens and beans, dairy, seafood and plant protein, total protein foods, and fatty acid ratio, were scored at 0 for the lowest and 5 or 10 for the highest consumption (with a score of 10 for fatty acids, whole grains, and dairy, and 5 for the other six components). Four moderation components containing sodium, refined grains, added sugars, and saturated fats scored 0 for the highest and 10 for the lowest consumption. The HEI total score was obtained from the sum of the scores of the 13 components, with a higher total score indicating higher diet quality [23]. The HEI scores categorized as ≤ 50%, 51−79%, and ≥ 80% are considered low, moderate, and good-quality diets, respectively [34]. Notably, energy-adjusted amounts of food items were used in calculating HEI scores using the residual method [35].

### Calculation of DQI-I

As described by Kim et al., the DQI-I is a method for evaluating diet quality in terms of four major dietary components: nutritional adequacy, moderation, variety, and overall balance [25]. Variety included two items containing the overall different food groups’ variety (fish and shellfish, meats and meat products, milk, and milk products, pulses and pulse products, eggs, grains, vegetables, and fruits with a score of 0–15) and the within-group protein source’s variety (fishes and shellfishes, meats and meat products, milk, and milk products, pulses and pulse products, and eggs with a score of 0–5). Adequacy contained adequate intake of fruits, vegetables, protein, fiber, grains, iron, vitamin C, and calcium (score of 0–40). In the moderation item, cholesterol, saturated fat, total fat, sodium, and empty calorie foods received negative scores (0–30). The overall balance was considered the fatty acid ratio and macronutrient ratio (score of 0–10). The total DQI-I score was calculated from the sum of the four component scores (score of 0-100), with a higher score showing a higher diet quality. Notably, energy-adjusted amounts of food items were used in calculating DQI-I scores using the residual method [35].

### Measurement of oxidative biomarkers

A morning urine sample (8–10 a.m.) was collected from all participants after 12 h of overnight fasting (for footballers, 72 h after the last exercise session). Enzyme-linked immunosorbent assay (ELISA) using commercial kits was performed to determine the concentrations of F_2a_-IP (8-epi PGF_2a_ kit, Abbexa, United Kingdom; catalog number: abx150311) and 8-OHdG (8-OHdG kit, Abbexa, United Kingdom; catalog number: abx150312). The calculation of F_2a_-IP and 8-OHdG concentrations was performed according to the product manual. Creatinine measurement in the urine using a spectrophotometry creatinine test kit (Pars Azmoon, Tehran, Iran) was utilized to normalize the values of F_2a_-IP and 8-OHdG.

### Statistical analysis

The Statistical Package for Social Sciences (SPSS Inc., Chicago, IL, USA; version 25) software was used to analyze the data, and *P* < 0.05 was considered statistically significant. The Kolmogorov-Smirnov test was performed to check the normality of the data, and normally distributed variables were presented as mean ± standard deviation (SD). The generalized estimating equation (GEE) model with an exchangeable correlation structure and identity link function was utilized due to the existing matching design in the data analysis. A suite GEE procedure was applied to compare the mean values of participants’ general characteristics and diet quality components in study groups, and to assess the differences in diet quality scores between the footballer group and the control group. Linear regression with GEE was conducted to determine the association between diet quality indices and oxidative biomarkers.

## Results

Since there were no dropouts in the present study, 90 people were involved in the current analysis. Table 1 outlines the mean values and the SD calculated for the general characteristics. The mean (SD) age of participants was 22.88 years (2.41), and the BMI was 22.08 kg/m^2^ (1.35). Footballers had lower levels of F_2a_-IP (pg/mg creatinine) (*P* < 0.001) and 8-OHdG (ng/mg creatinine) (*P* < 0.001) compared to the control group.

**Table 1:** Characteristics of the study participants

Variables	Footballers (*n* = 45)	Controls (*n* = 45)	All participants (*n* = 90)	*P*-value*
Age (years)	22.89 ± 2.42	22.87 ± 2.42	22.88 ± 2.41	0.96
BMI (kg/m^2^)	22.06 ± 1.34	22.09 ± 1.37	22.08 ± 1.35	0.09
Weight (kg)	67.85 ± 7.03	66.80 ± 10.41	67.33 ± 8.85	0.47
Height (cm)	175.18 ± 6.63	173.22 ± 10.63	174.2 ± 8.86	0.29
8-OHdG (ng/mg creatinine)	10.90 ± 3.66	20.30 ± 9.52	15.6 ± 8.59	**< 0.001**
F_2a_-IP (pg/mg creatinine)	124.51 ± 41.46	241.6 ± 112.38	183.06 ± 102.76	**< 0.001**

BMI, body mass index; 8-OHdG, 8-hydroxy-2-deoxy guanosine; F_2a_-IP, F_2a_-isoprostane

* *P*-values were determined by the GEE model

-Values are mean ± SD.

- Significant values are shown in bold

The HEI and DQI-I total scores and their components are presented in Table 2. The footballers’ HEI total score (*P* = 0.001) and DQI-I total score (*P* = 0.001) were significantly higher than those of the control group. Among the HEI components, total fruit (cup/day) (*P* = 0.03), whole grains (g/day) (*P* = 0.03), dairy (cup/day) (*P* = 0.002), and total protein foods (g/day) (*P* = 0.002) were significantly greater in the footballer group. In contrast, saturated fat (% energy) intake (*P* < 0.001) was higher in the control group. Moreover, the footballer group exhibited significantly higher scores in variety (*P* = 0.03), adequacy (*P* < 0.001), and overall balance (*P* = 0.01) among the DQI-I components.

**Table 2:** Components of diet quality indices

Variables	Footballers (*n* = 45)	Controls (*n* = 45)	All participants (*n* = 90)	*P*-value*
**HEI Components**				
Total Fruits (cup/day)	0.74 ± 0.34	0.60 ± 0.28	0.67 ± 0.32	**0.03**
Whole Fruits (cup/day)	0.70 ± 033	0.59 ± 0.27	0.64 ± 0.31	0.06
Total Vegetables (cup/day)	1.28 ± 0.38	1.20 ± 0.33	1.24 ± 0.35	0.25
Greens and Beans (cup/day)	0.31 ± 0.11	0.32 ± 0.12	0.32 ± 0.11	0.86
Whole Grains (g/day)	32.42 ± 21.30	22.74 ± 18.34	27.58 ± 20.35	**0.03**
Dairy (cup/day)	0.45 ± 0.17	0.35 ± 0.13	0.40 ± 0.16	**0.002**
Total Protein Foods (g/day)	81.31 ± 14.33	72.46 ± 12.20	76.88 ± 13.96	**0.002**
Seafood and Plant proteins (cup/day)	0.177 ± 0.04	0.171 ± 0.04	0.17 ± 0.04	0.51
Fatty Acid Ratio	2.27 ± 0.37	2.34 ± 0.28	2.30 ± 0.33	0.34
Refined Grains (g/day)	172.38 ± 36.01	175.76 ± 38.41	174.07 ± 37.06	0.63
Added Sugars (% energy)	6.60 ± 2.38	6.74 ± 2.88	6.67 ± 2.63	0.82
Sodium (g/day)	2.71 ± 0.43	2.81 ± 0.57	2.76 ± 0.50	0.36
Saturated Fats (% energy)	8.44 ± 1.05	9.45 ± 1.09	8.95 ± 1.18	**< 0.001**
HEI total score	60.48 ± 4.83	56.64 ± 5.60	58.56 ± 5.55	**0.001**
**DQI-I Components**				
Variety-food groups and protein sources	19.88 ± 0.74	19.44 ± 1.13	19.66 ± 0.98	**0.03**
Adequacy	38.53 ± 1.30	36.42 ± 2.47	37.47 ± 2.23	**< 0.001**
Moderation	6.66 ± 3.31	6.60 ± 2.90	6.63 ± 3.09	0.92
Overall Balance	5.09 ± 0.98	4.55 ± 1.02	4.82 ± 1.03	**0.01**
DQI-I total score	70.17 ± 3.94	67.01 ± 3.87	68.59 ± 4.20	**0.001**

HEI, healthy eating index; DQI-I, diet quality index-international

* *P*-values were determined by the GEE model

-Values are mean ± SD.

- Significant values are shown in bold

The comparison of diet quality differences between the two groups is shown in Table 3. The footballer group had significantly higher scores in the HEI total score (β = 4.01; *P =* 0.003) and DQI-I total score (β = 3.09; *P =* 0.005) than the control group.

**Table 3:** Comparison of the HEI and DQI-I between the footballer and control groups

Variables		B	SE	95% Wald confidence interval (lower, upper)	*P*-value*
HEI	Controls	Reference	-	-	-
	Footballers	4.01	1.35	(1.35, 6.66)	**0.003**
DQI-I	Controls	Reference	-	-	-
	Footballers	3.09	1.11	(0.92, 5.27)	**0.005**

B, regression coefficient; SE, standard error; HEI, healthy eating index; DQI-I, diet quality index-international

* *P*-values were determined by the GEE model

- Significant values are shown in bold

The linear regression analysis for the relationship between diet quality and 8-OHdG in all participants is presented in Table 4. The results showed significant negative associations; as HEI and DQI-I increased by one unit, 8-OHdG decreased by 0.29 (*P* = 0.04) and 0.46 (*P =* 0.005), respectively.

**Table 4:** Association of the HEI and DQI-I with 8-OHdG in all participants

Variables		B	SE	95% Wald confidence interval (lower, upper)	*P*-value*
HEI	Model 1^a^	-0.31	0.15	(-0.6, -0.01)	**0.03**
	Model 2^b^	-0.29	0.14	(-5.81, -0.006)	**0.04**
DQI-I	Model 1^a^	-0.61	0.18	(-0.96, -0.25)	**0.001**
	Model 2^b^	-0.46	0.16	(-0.78, -0.14)	**0.005**

B, regression coefficient; SE, standard error; HEI, healthy eating index; DQI-I, diet quality index-international

* *P*-values were determined by linear regression analysis using the GEE model

^a^ Crude model

^b^ Adjusted for physical activity

- Significant values are shown in bold

Table 5 indicates the results of the linear regression analysis for the association between diet quality and F_2a_-IP in all participants. HEI and DQI-I were significantly and negatively associated with F_2a_-IP; as HEI and DQI-I increased by one unit, F_2a_-IP decreased by 3.53 (*P* = 0.04) and 5.53 (*P* = 0.004), respectively.

**Table 5:** Association of the HEI and DQI-I with F_2a_-IP in all participants

Variables		B	SE	95% Wald confidence interval (lower, upper)	*P*-value*
HEI	Model 1^a^	-4.30	1.92	(-8.06, -0.53)	**0.02**
	Model 2^b^	-3.53	1.76	(-7.00, -0.06)	**0.04**
DQI-I	Model 1^a^	-7.36	2.13	(-11.53, -3.18)	**0.001**
	Model 2^b^	-5.53	1.91	(-9.29, -1.77)	**0.004**

B, regression coefficient; SE, standard error; HEI, healthy eating index; DQI-I, diet quality index-international

* *P*-values were determined by linear regression analysis using the GEE model

^a^ Crude model

^b^ Adjusted for physical activity

- Significant values are shown in bold

## Discussion

This study was designed to compare diet quality indices between two groups of football athletes and active controls and investigate their relationship with urinary OS parameters. The results demonstrated that the DQI-I and HEI were higher in the footballer group. Furthermore, the DQI-I and HEI were negatively related to urinary OS parameters.

As mentioned earlier, the HEI is a method to examine dietary patterns and their health consequences [23]. This index was created by the USDA to ascertain the overall quality of the diet and clarify whether dietary habits in populations align with specified guidelines or not [36]. The HEI scores in the current study showed moderate diet quality in both groups. However, the footballers’ HEI scores were higher than those of the control group.

In a study, Beba et al. investigated HEI and its relationship with body composition in Iranian football players and referees. Their results showed that HEI was significantly correlated with the percentage of muscle mass, WHR, and total body fat (TBF) in male referees and football players. Additionally, in the study mentioned above, consistent with the present study, the HEI scores showed moderate quality of the diet [21]. Therefore, encouraging football athletes to improve their diet quality can be considered.

Regarding HEI components, the intake of whole grains, total protein foods, total fruit, and dairy products was higher in the footballers than in the controls. Furthermore, saturated fat intake in the footballers was lower than in the controls. In this regard, a study by Georgiou et al. indicated that in young women who exercise regularly, the intake of legumes and fruits is higher, and the intake of fats is lower than in those who do not exercise [37]. Additionally, a study by Xu et al. indicated that HEI scores were significantly correlated with physical performance, such as power knee extensor and gait speed [38]. This association seems to be due to the antioxidants found in fruits and vegetables [39]. Although antioxidant supplementation has been suggested to improve performance and facilitate faster recovery in intense exercise, the theoretical basis for why antioxidants might enhance performance is unclear [39]. As a result, a healthy, high-quality diet may likely lead to improved athletic performance.

In addition, the results demonstrated a negative association between HEI and levels of 8-OHdG and F_2a_-IP. A systematic review conducted by Aleksandrova et al. proved that following the HEI diet was correlated with lower concentrations of F_2a_-IP, and the higher the HEI scores, the lower the OS [40]. Greater adherence to HEI may likely lead to increased blood antioxidants, redox, and inflammatory balance [18]. However, one study showed no relationship between HEI and OS parameters [41]. To justify this, it can be said that the consumption of vitamins A and E in their studied samples was low, both of which play a substantial role in eliminating free radicals [41, 42].

The DQI-I represents a useful index for assessing diet quality across countries [25]. The results indicated that the DQI-I was higher in the footballers than in the controls. Furthermore, the DQI-I had a negative association with urinary 8-OHdG and F_2a_-IP levels, which could be explained by the consumption of vitamin C, vegetables, and fruits as the main sources of dietary antioxidants [43], considered to be the principal components of this dietary index. Vegetables, fruits, and fruit juices are good sources of vitamin C [44]. It is noteworthy that based on the results, the DQI-I, compared to the HEI, exerted a greater effect on decreasing F_2a_-IP and 8-OHdG levels. Through fruit, vegetable, and vitamin C intake, the body’s oxidant status will be improved, and the formation of F_2a_-IP and 8-OHdG will be decreased. To our knowledge, little research on the relationship between DQI-I and OS has been performed, which makes it difficult to compare the results of the present study with similar studies. Only a study by Kim et al. found that diet quality may affect the reduction of OS (measured by malondialdehyde). At the same time, this research proved no correlation between DQI-I and 8-OHdG [13].

### Limitations and strengths

The current study has some limitations. Although the FFQ is the most applicable tool for obtaining the long-term usual intake of subjects, recall bias is inevitable. Nevertheless, an attempt was made to solve this problem through face-to-face interviews using a skilled nutritionist. Moreover, using the IPAQ questionnaire may cause misreporting bias due to respondents’ memory errors. Although some confounding factors were adjusted, the recruitment of control participants from medical university students may be considered a limitation due to the potential of having higher nutritional knowledge. In addition, there may be other confounding factors that were not considered in the present study. Another limitation is that because of the cross-sectional nature of the present research, inferring causality is impossible. Thus, in future studies, it is recommended to investigate the mechanism of the effect of the evaluated dietary indicators on the OS situation. Also, the present study sample size was small.

There are several strengths in the current study. First, to our knowledge, this is the first study that evaluated DQI-I and HEI and their relationship with oxidative levels in football athletes. Second, we used the DQI-I and HEI indices, which include key components in the Iranian dietary guidelines (such as the intake of whole grains, vegetables, fruits, and sodium) [45]. Third, a valid FFQ was used to assess food intake.

## Conclusions

In the present study, the quality of the footballers’ diet was higher than that of the control group. The diet quality indices of HEI and DQI-I revealed significant and negative associations with OS, as measured by 8-OHdG and F_2a_-IP, indicating that consuming a high-quality diet rich in vegetables and fruits as the main sources of dietary antioxidants may reduce OS. Further research must be conducted to generalize the findings to other groups and confirm the present findings.

## Data Availability

The data sets used and/or analyzed during the current study are available from the corresponding author on reasonable request.
